# Orbital Blowout Fracture From Nose Blowing

**DOI:** 10.5811/cpcem.2016.11.30820

**Published:** 2017-01-17

**Authors:** Mohammad R. Mohebbi, Cory M. Shea

**Affiliations:** University of Missouri, Department of Emergency Medicine, Columbia, Missouri

A 38-year-old woman with a history of seasonal allergies presented to the emergency department with sudden onset of left periorbital swelling following nose blowing. There was no history of trauma, prior surgery, sinusitis or associated illness. On examination there was significant non-tender left periorbital swelling, with crepitus on palpation (Image 1). Extraocular movements, pupillary reflexes, fundoscopic examination and visual acuity were normal. Maxillofacial computed tomography (CT) showed subcutaneous emphysema (Image 2) and spontaneous left orbital floor blowout fracture with herniation of the orbital fat, and inferior rectus muscle and inferior oblique muscle into the left maxillary sinus (Image 3). Upon reevaluation, extraocular movements were normal. The patient was referred for oculoplastics evaluation.

Orbital emphysema is usually secondary to traumatic orbital fracture. In the absence of trauma, this is a rare condition. 1 Other mechanisms, including infection, pulmonary barotrauma, injury from compressed-air hoses, and complications from surgery and sneezing, have been reported to cause orbital emphysema.^2^ Blow-out orbital fracture in absence of trauma is a rare condition as described in our patient. A few cases of orbital floor fracture following forceful nose blowing have been reported in the literature.^3^,^4^ Blow-out orbital fractures most often involve the thinnest portions of the orbit, namely inferior (36.7%) and medial (31%) orbital wall.^2^,^5^ A proposed mechanism for nontraumatic orbital fracture is that chronic maxillary sinusistis may weaken the orbital floor, making it more vulnerable to fracture by increased pressure following forceful nose blowing.^4^ Patients should be instructed to avoid nose blowing, coughing or the Valsalva maneuver for at least two weeks after the injury.^6^ There are many studies detailing the use of antibiotics in maxillofacial fractures, but very few on the use of antibiotics in isolated orbit fractures.^7^ Patients with preexisting sinus disease may be at an increased risk for infection.^7^ Prophylactic oral antibiotics (such as amoxicillin-clavulanate or azithromycin) to cover sinus pathogens are generally recommended for patients with orbital fracture into a sinus.^8^

In conclusion, emergency physicians should consider nontraumatic orbital fracture in patients with orbital emphysema with no history of trauma. CT is the recommended study of choice.

## Figures and Tables

**Image 1:**
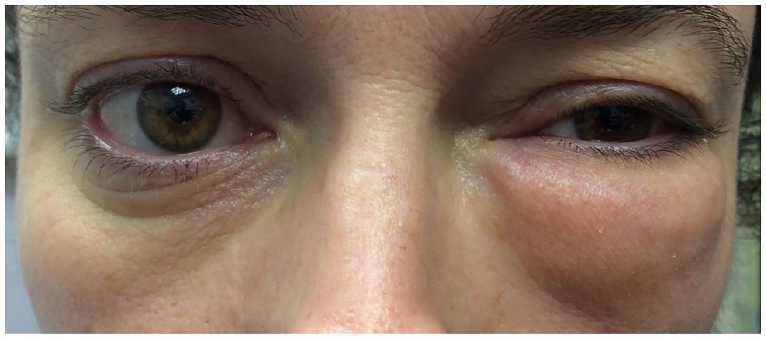
Non-tender left periorbital swelling.

**Image 2:**
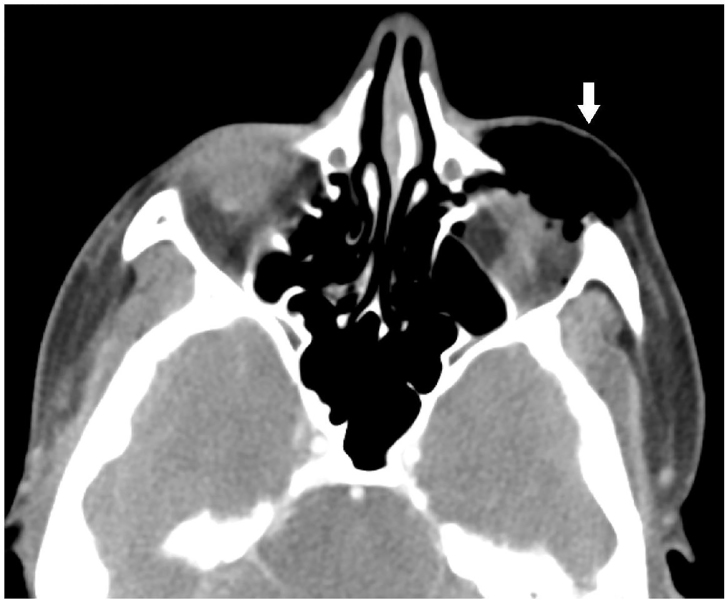
Computed tomography shows subcutaneous emphysema around the left orbit (arrow).

**Image 3:**
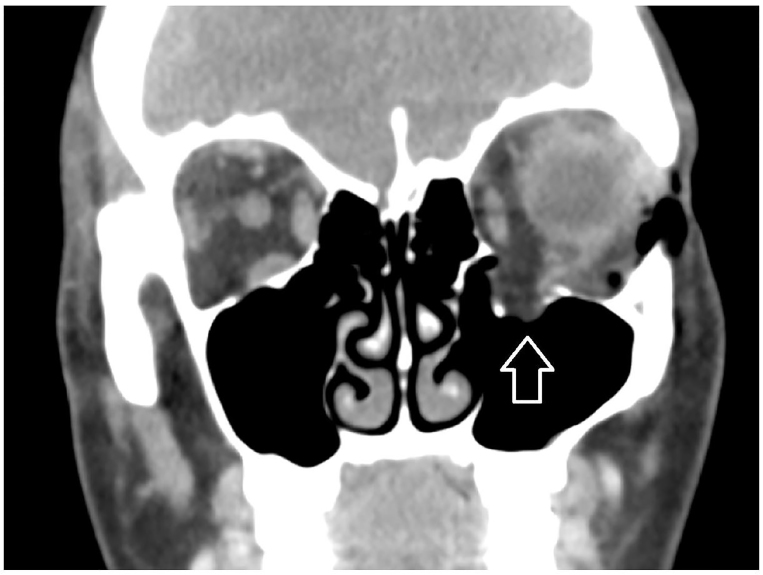
Computed tomography shows left orbital floor blowout fracture with herniation of the orbital fat, inferior rectus muscle and inferior oblique muscle into the left maxillary sinus (arrow).
